# Ferrite Shield to Enhance the Performance of Optically Pumped Magnetometers for Fetal Magnetocardiography

**DOI:** 10.3390/jcm12093078

**Published:** 2023-04-24

**Authors:** Gabriela P. Tardelli, Tan Phan, Janette Strasburger, Oswaldo Baffa, Ronald Wakai

**Affiliations:** 1 Department of Medical Physics, Wisconsin Institute for Medical Research, University of Wisconsin-Madison, Madison, WI 53705, USA; tardelli@wisc.edu (G.P.T.); tphan8@wisc.edu (T.P.); 2 Department of Physics, School of Philosophy, Science and Letters at Ribeirão Preto, University of São Paulo, Ribeirão Preto 14040-900, SP, Brazil; baffa@usp.br; 3 Division of Cardiology, Department of Pediatrics, Children’s Hospital of Wisconsin-Milwaukee, Milwaukee, WI 53226, USA; jstrasburger@childrenswi.org

**Keywords:** magnetic shielding, ferrite, fetal magnetocardiography, optically pumped magnetometers, arrhythmias

## Abstract

Fetal magnetocardiography (fMCG) has proven to be an important tool for the prenatal monitoring of electrical cardiac activity; however, the high cost of superconducting quantum instrumentation (SQUID) poses a limitation for the dissemination of fMCG as a routine clinical technique. Recently, optically pumped magnetometers (OPMs) operating within person-sized, cylindrical shields have made fMCG more practical, but environmental magnetic interference entering through the shield opening substantially degrades the quality of fMCG signals. The goal of this study was to further attenuate these interferences by placing the OPM array within a small ferrite shield. FMCG recordings were made with and without the ferrite shield in ten subjects inside a person-sized, three-layer mu-metal cylindrical shield. Although the fetal signal was slightly attenuated, the environmental interference was reduced substantially, and maternal interference was also diminished. This increased the signal-to-noise ratio significantly and improved the resolution of the smaller waveform components. The performance improvement was highest in the axial direction and compensated for a major weakness of open-ended, person-sized shields. The ferrite shield is especially beneficial for the deployment of triaxial OPM sensors, which require effective shielding in all directions.

## 1. Introduction

Fetal magnetocardiography (fMCG) is a noninvasive modality that records the magnetic activity of the fetal heart during pregnancy. It has emerged as an important complementary diagnostic tool to fetal echocardiography, which records mechanical activity and blood flow, and an alternative to fetal electrocardiography, which records electrical activity but is not used clinically due to its poor signal quality. Numerous studies have shown that fMCG can be recorded reliably throughout the last half of pregnancy and provides valuable information, including assessments of fetal heart rate, beat-to-beat fetal heart rate variability, fetal activity, and fetal rhythm. Clinically, the most important application of fMCG is the diagnosis of fetal arrhythmia [1,2]. The use of fMCG for this application was endorsed by the American Heart Association in its inaugural statement on diagnosis and treatment of fetal cardiac disease [3].

Recently, fMCG technology has been revolutionized by the emergence of optically pumped magnetometers (OPM) [4,5,6], a cryogen-free sensor with sensitivity similar to that of superconducting quantum interference devices (SQUIDs), which are the sensors currently in use [7,8,9]. In addition to reduced cost, size, and complexity, OPM-based systems enable the use of smaller magnetic shields. Person-sized cylindrical magnetic shields have been demonstrated as cost-effective alternatives to magnetically shielded rooms (MSRs) [10,11]; however, to ensure patient comfort and avoid the risk of claustrophobia, one end of the cylindrical shield is left open. This results in substantially reduced shielding performance toward the open end of the shield.

Several methods have been investigated to improve the shielding performance of open-ended, person-sized shields. It is well known from simulations that lengthening the shield improves performance; however, this increases the cost and difficulty of finding suitable space in a clinical setting. Adding a fourth layer of shielding to a typical three-layer, person-sized shield can result in performance similar to that of a large-size MSR [10], but again increases the cost and size of the shield. Another approach implemented by several groups [12,13] is to add an extension that reduces the diameter of the opening to approximately 60 cm. This was demonstrated in simulations to improve the shielding by a factor of two and shift the optimal position towards the center of the shield [14]. For fMCG, however, it is not feasible to appreciably narrow the opening. The diameter of the inner shield needs to be 75 cm or greater to allow a pregnant woman to comfortably enter. Lastly, the best shielding performance is achieved with superconducting shields [15], but the cost at the moment exceeds that of an MSR.

The problem of leakage interference from the shield opening is considerably more severe for fMCG than for other applications. For magnetoencephalography (MEG) and even MCG, the sensors can be positioned much closer to the closed than the open end of the shield. For fMCG, however, the sensors are positioned near the middle of the shield. The leakage interference is especially strong in the longitudinal direction because, for cylindrical geometries, the shielding factor is approximately ten times higher in the transverse direction than in the longitudinal. In the study by Strand and coworkers, the sensors were oriented to record only the transverse components of the fMCG signal due to the low signal-to-noise ratio (SNR) of the longitudinal component [11].

In this study, we investigated the use of a small ferrite shield to augment the performance of a conventional person-sized magnetic shield. Small ferrite shields have been used previously by Kornack and coworkers to circumvent the Johnson noise associated with mu-metal shields [16]. To our knowledge, this is the first use of ferrite shields for a biomagnetism application. We show that the method is practical and provides a major benefit by allowing the longitudinal component of the fMCG to be recorded with SNR similar to that of the transverse components.

## 2. Materials and Methods

### 2.1. Instrumentation

**Figure 1:**
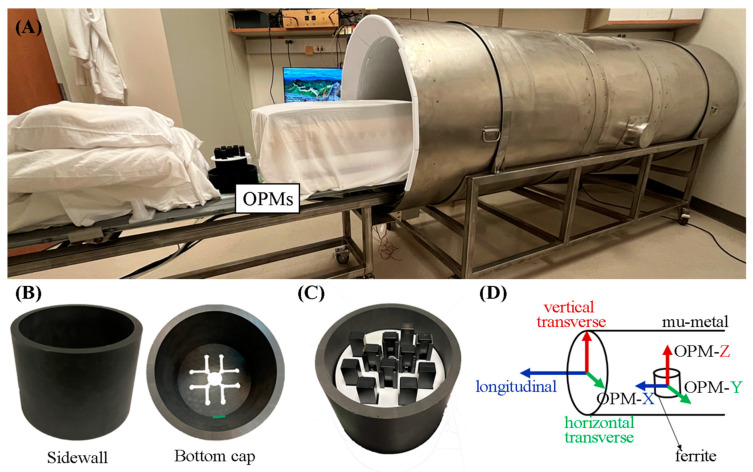
(**A**) Photograph showing the open-ended, 3-layer mu-metal cylindrical shield, the sliding patient table, and the OPM sensors. (**B**) Photograph of the side wall and bottom cap of the ferrite shield showing the sensors’ access hole. (**C**) Photograph of the sensors distributed within the ferrite shield. (**D**) Diagram showing the directions with respect to the mu-metal shield. The longitudinal direction is defined by the long axis of the cylinder. The vertical transverse direction is perpendicular to the floor, and the horizontal transverse direction is parallel to the floor. The OPMs were oriented so that their Y and Z outputs record the magnetic field in the horizontal and vertical transverse directions, respectively, and the X output (Gen3 sensors) records the magnetic field in the longitudinal direction.

The measurements were performed within a cylindrical three-layer mu-metal shield (Amuneal Inc., Philadelphia, PA, USA) with an inner diameter of 75 cm and length of 2.5 m (Figure 1A). One end of the shield was closed, and the other was kept open to avoid the risk of claustrophobia. It was placed on a custom-made support with a detachable extension that allows the patient’s table to slide in and out of the shield.

The study took place at the Wisconsin Institute for Medical Research at the University of Wisconsin–Madison, where the earth’s magnetic field is ≅ 54 μT. The residual field was reduced to approximately 10 nT by placing the shield perpendicular to the earth’s magnetic field. A ferrite cylindrical shield (height: 12.5 cm; inner diameter: 15 cm; and thickness: 1 cm), depicted in Figure 1B, was placed inside the mu-metal shield with its axis perpendicular to the mu-metal shield’s axis. The ferrite shield had one end open and the other closed with a removable end cap of the same material and thickness. The end cap had 12 access holes in it, allowing the cables to exit.

Three OPM sensor versions—Gen1, Gen2, and Gen3 (QuSpin Zero Field Magnetometer, QuSpin Inc., Louisville, CO, USA)—were used to record the fMCG. The Gen1 and Gen2 sensors were dual-axis sensors with an intrinsic magnetic field resolution of 10–15 fT/(Hz)^1/2^. Each sensor measures two orthogonal components of the magnetic field, one parallel to the long axis of the sensor (Z) and the other parallel to the short axis (Y). The Gen2 sensors were smaller than the Gen1 sensors. The Gen3 sensors became available toward the end of the study. They are triaxial sensors (Z, Y, and X) and have a slightly lower magnetic field resolution. A 3D-printed plastic holder accommodated 11 OPM sensors arranged in an offset square grid pattern of 9 × 9 cm (Figure 1C). The sensors were oriented so that the Z and Y outputs recorded the magnetic field in the horizontal and vertical transverse directions of the mu-metal shield (Figure 1D), and the X component of the triaxial sensors recorded the magnetic field in the longitudinal direction. The signals were digitized at 1 kHz using a LabView data acquisition system (National Instruments, Austin, TX, USA). Signal processing was performed using a custom computer program written in Matlab (MathWorks Inc., Natick, MT, USA).

### 2.2. Vertical Placement of Sensor Array within Ferrite Shield

The vertical placement of the sensor array within the ferrite shield affects the shielding effectiveness and signal fidelity. If the sensors are positioned well below the top of the shield, the interference will be strongly attenuated but the signal may also be diminished. If the sensors are positioned near the top of the shield, the signal loss will be minimal, but the shielding effectiveness will also be reduced. To help determine an optimal placement, we measured the shielding factor as a function of the vertical position of the sensor array with respect to the top of the ferrite shield, using an external magnetic coil to simulate environmental interference. The shielding factor is defined as the ratio of the magnetic field in the absence of the ferrite shield to the field in the presence of the shield.

### 2.3. Attenuation of Environmental Interference

The effectiveness of the ferrite shield in attenuating environmental interference was assessed by placing the center of the OPM array 140 cm from the shield opening and recording the interference with and without the ferrite shield. Five dual-axis and four tri-axis OPMs were used to obtain the noise in all three directions. Each measurement lasted 30 s. The power spectra for each direction in both cases were obtained using Welch’s method in Matlab.

### 2.4. Human Studies

#### 2.4.1. Subjects

The protocol was approved by the UW-Madison Health Sciences IRB, and informed consent was obtained from all subjects. The subjects were 10 adult pregnant women, studied at 21–36 weeks of gestation. Seven of the pregnancies were uncomplicated. One was complicated by fetal long QT syndrome (LQTS). A second was complicated by endocardial fibroelastosis and fetal AV block associated with isoimmune disease. A third was a case of congenital heart disease, tetralogy of Fallot.

#### 2.4.2. Data Collection

The data were collected with the mother lying prone on two sections of foam mattress separated by a gap. The sensor array was covered by a thin piece of foam and placed within the gap in contact with the mother’s abdomen from below. Airflow was applied to the sensors to prevent them from overheating. After positioning the mother, the patient table was slid into the mu-metal shield for data collection. The sensor array was approximately 140 cm from the mu-metal shield opening. Two 60 s runs were recorded—one with and one without the ferrite shield.

#### 2.4.3. Data Processing

The performance of the ferrite shield was assessed using four parameters: the amplitude of the fetal and maternal QRS complexes (fQRS and mQRS, respectively), the amplitude of the environmental noise, and the SNR. The parameters were calculated separately for each sensor and direction.

To analyze the effect of the shield, the parameters must be measured from the raw signal. Thus, a 1–100 Hz band-pass filter was applied to remove the offset without removing the main interferences. The amplitude of each fQRS and mQRS was measured as the peak-to-peak amplitude. The environmental noise amplitude was defined as the root-mean-square of each interval between the end of one QRS complex and the beginning of the next. After excluding artifacts, all of the fQRS, mQRS, and noise amplitudes throughout the signal were averaged, yielding one final value for each variable. Finally, the SNR was calculated as the average fQRS amplitude divided by the average noise amplitude.

#### 2.4.4. Statistical Analysis

We performed an ANOVA with a 2 × 2 factorial randomized block design where the patients were considered blocks (random effects). The presence of the shield and the direction, as well as the interaction between them, were considered fixed effects. All analyses were performed using the mixed procedure of SAS.

## 3. Results

### 3.1. Vertical Placement of Sensor Array within Ferrite Shield

**Figure 2:**
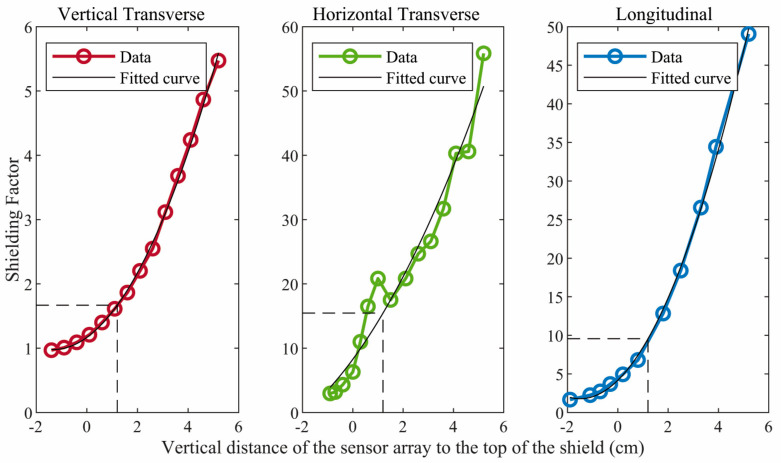
Shielding factor in each direction for different vertical positions of the sensor array with respect to the top/open end of the ferrite shield. Negative values indicate that the sensor array was above the top of the shield. The data were fitted in a polygonal curve and the shielding factors obtained for the vertical transverse, horizontal transverse, and longitudinal directions were 1.7, 15.5, and 9.6, respectively.

Figure 2 shows the shielding factor in each direction as a function of the vertical position of the sensor array with respect to the top of the ferrite shield. The shielding factors are modest in the transverse directions but substantial in the longitudinal direction. Although the shielding factors increase when the sensors are positioned farther within the shield, an additional, critically important consideration is the need to situate the sensors as close as possible to the fetal heart. This is difficult unless the sensors are near the top of the shield. Based on these considerations, we chose to position the OPM sensors so that they were approximately 1.2 cm below the top of the ferrite shield. At this position, the shielding factors are approximately 1.7, 15.5, and 9.6, respectively, in the vertical transverse, horizontal transverse, and longitudinal directions.

### 3.2. Attenuation of Environmental Interference

**Figure 3:**
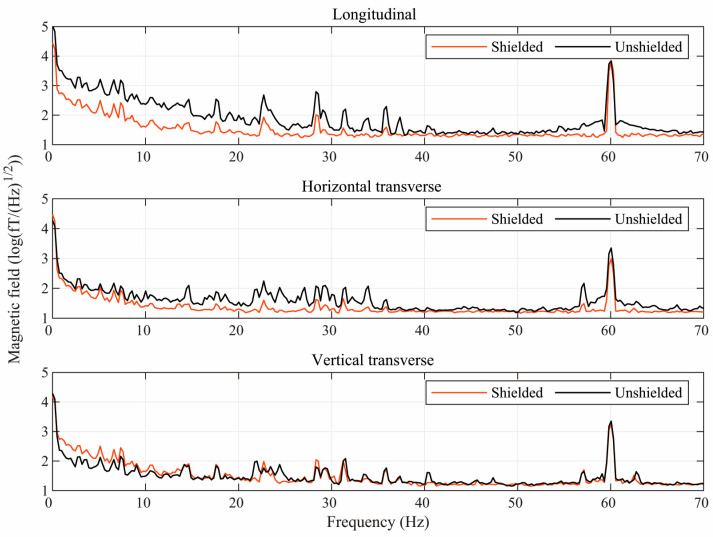
Semilog plots of the power spectra of the environmental magnetic noise in three directions inside the mu-metal shield with (red) and without (black) the ferrite shield around the sensors.

Figure 3 shows the effect of the ferrite shield on the power spectra of the environmental magnetic noise components near the center of the mu-metal shield. As expected, in the absence of the ferrite shield, the longitudinal direction is much noisier than both transverse directions. When using the ferrite shield, the interference in all directions is significantly attenuated, but the degree of attenuation is much greater for the longitudinal direction. Thus, the ferrite shield compensates for the poorer performance of the mu-metal shield in the longitudinal direction so that the residual interference is similar in all directions.

### 3.3. Human Studies

Analysis and interpretation of the human data were complicated by several factors. The data show occasional inconsistencies and anomalies, such as greater-than-expected changes in the signal amplitude between the shielded and unshielded data. We largely attribute this to the fact that removing the shield requires repositioning the subject, and changes in the position and orientation of the fetus between measurements can result in inconsistencies. furthermore, for reasons we do not completely understand, maternal interference was absent in both shielded and unshielded data for two patients and was present in the shielded data but absent in the unshielded data for one patient. Statistical analysis was performed only in the five cases where maternal interference was present in both unshielded and shielded data.

Table 1 presents the results of the statistical analyses for the transverse directions. The analysis could not be performed for the longitudinal direction because the environmental interference was so strong that the unshielded signal could not be resolved. Using the ferrite shield affected all parameters (*p* < 0.001): the fQRS and mQRS amplitudes and the noise amplitude were reduced considerably, while the SNR increased significantly. The fQRS amplitude was reduced by 47% in the horizontal transverse direction and 32% in the vertical transverse direction, the mQRS amplitude was reduced by 47% and 29%, the noise was reduced by 55% and 47%, and the SNR increased by 32% and 24%, respectively. The direction did not affect the parameters, but the interaction between the presence of the shield and the direction was significant for the fQRS amplitude (*p* = 0.034). The multiple comparisons showed that after including the shield, the difference in the horizontal and vertical transverse fQRS amplitudes became significant (*p* = 0.033).

**Figure 4:**
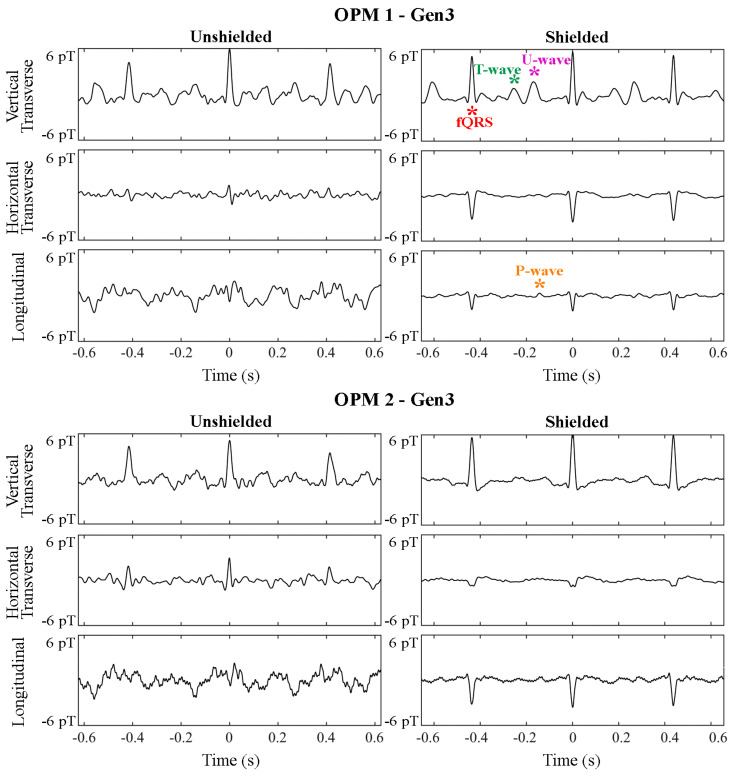
Effect of the ferrite shield in all components of two triaxial (Gen3) OPMs in fMCG recordings of a fetus with tetralogy of Fallot in sinus rhythm. The strips are 1.2 s in duration and depict an average of 5 s of fMCG. A signal filter was applied prior to averaging. Components of the fetal signal are depicted by red (QRS), green (T-wave), magenta (U-wave), and orange (P-wave) asterisks.

The effectiveness of the ferrite shield is clearly seen in the fMCG traces shown in Figure 4. Without the shield, the longitudinal component is completely dominated by noise. Although it is not possible to quantitatively compare the effect of the ferrite shield on longitudinal parameters, the improvement is evident when comparing the traces.

**Figure 5:**
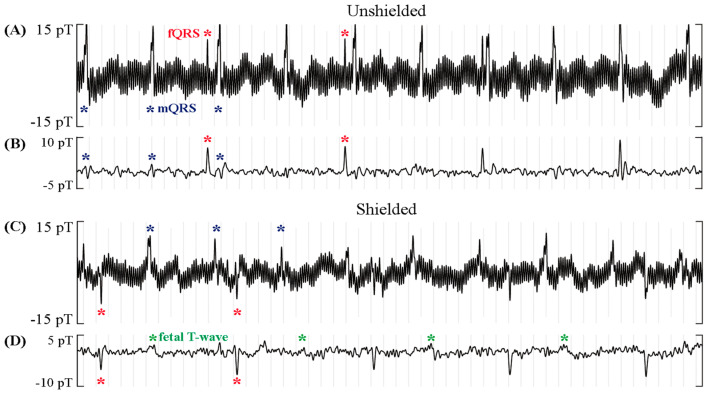
Effect of the ferrite shield around the OPMs in fMCG recordings of a fetus with complete heart block and mother with high heart rate. The rhythm strips are 5 s in duration. A significant reduction in maternal QRS and noise amplitude (blue asterisks) and a slight reduction in fetal QRS (red) are observed in the raw rhythm in (**A**,**C**). The filtered rhythm in (**B**,**D**) shows that, with the ferrite shield, the maternal interference was completely gone, and the fetal T-wave (green) was resolved.

In the rhythm strips in Figure 5, the fetus presents with a slow fetal heart rate due to a complete heart block, while the mother shows a relatively high heart rate. Figure 5A,B show raw and signal-processed fMCG recordings obtained without the ferrite shield, while Figure 5C,D show recordings made with the ferrite shield. The results in Table 1 are compatible with the rhythm strips in Figure 5. The raw fMCG shows that the amplitudes of the fQRS, mQRS, and interference were reduced by the ferrite shield. The processed fMCG shows that, after applying signal processing to remove interferences, fetal T-waves (green asterisks) could be resolved with the ferrite shield. Furthermore, maternal interference was completely removed by the ferrite shield but was visible (blue asterisks) in the unshielded fMCG tracings.

**Figure 6:**
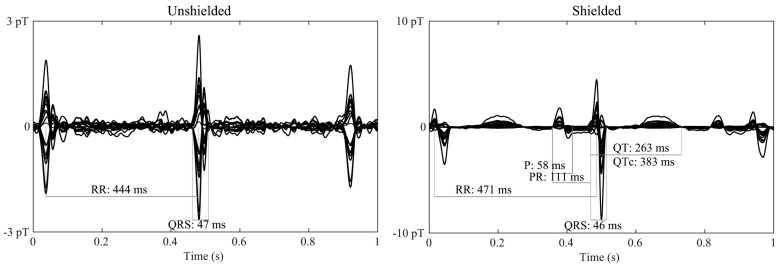
Average waveform of fMCG recordings of a normal fetus in the absence (**left**) and presence (**right**) of the ferrite shield around the array of OPMs. The waveform displayed has gone through signal processing. The presence of the ferrite shield improves the resolution of the smaller fMCG components (P- and T-waves).

Figure 6 shows averaged waveforms from fMCG recordings of a healthy fetus. Although signal-processing techniques were applied to obtain the final waveform, it is evident that the SNR improved with the use of the ferrite shield, and the P- and T-wave components of the fMCG were better resolved.

## 4. Discussion

In this study, we showed that placing an OPM array within a small ferrite shield can significantly improve the performance of OPM-based fMCG systems that use person-sized magnetic shields. The improvement is the result of the ferrite shield overcoming a main weakness of cylindrical mu-metal shields, namely, the low shielding factor along the longitudinal direction. As expected, the performance improvement is modest in the transverse directions but substantial in the longitudinal direction. For this reason, the shield is especially helpful when using triaxial OPM sensors, which have recently become commercially available. These sensors allow all three components of the magnetic signal to be recorded at the same location. In general, the signal components are independent, and it is necessary to measure all three to extract all the information available in the signal. This is problematic when using cylindrical, person-sized shields due to the low shielding factor in the longitudinal direction; however, as demonstrated here, the ferrite shield can compensate for this shortcoming, allowing all three channels of the triaxial output to exhibit a similarly high SNR. In a prior study utilizing dual-axis OPMs, we oriented the sensors to measure only the transverse components. This provided signals with a high SNR at the expense of the measurement of the longitudinal signal component. When using dual-axis OPMs, the ferrite shield allows flexible placement of the sensors, enabling both the transverse and longitudinal signal components to be measured. This provides a more complete characterization of the signal.

In some subjects, the increase in the signal quality resulting from use of the ferrite shield provided significantly improved resolutions of the P- and T-waves. These small waveform components are crucial to rhythm assessment. The differential diagnosis of most bradycardias and supraventricular tachycardias is based on the relative timing of the atrial and ventricular activations represented by the P-wave and QRS complex. Detection of repolarization abnormalities, such as QTc prolongation in long QT syndrome or ST segment changes due to ischemia, requires resolution of the T-wave. Fetuses with congenital heart disease may also benefit from the fMCG evaluation because arrhythmia is a major cause of morbidity and mortality in this population, although arrhythmia was not present in the fetus studied here with tetralogy of Fallot. The technical improvements demonstrated in this study can provide a meaningful increase in diagnostic capability and may allow fMCG to be performed at earlier gestational ages.

A particular advantage of our method is the attenuation of maternal interference, which is the dominant interference in fMCG recordings. Typically, it is not possible to shield the sensors from maternal interference and other biological interferences emanating from the subject. Instead, signal processing is relied upon. The most effective methods utilize spatial filtering, such as independent component analysis (ICA). In many cases, however, ICA cannot cleanly separate the fetal and maternal signals because their spatial characteristics, i.e., signal topographies, are not sufficiently distinct. This results in the loss of the fetal signal or the incomplete removal of maternal interference, depending on the design of the filter. The ferrite shield can mitigate this problem by attenuating maternal interference and/or altering its spatial characteristics to improve the ability of the spatial filter to distinguish it from the fetal signal.

An additional advantage of the shield is improved signal visualization during data acquisition. While signal processing is highly effective in improving the signal resolution, it cannot be performed in real time. In addition to significant computation time, many signal-processing methods require user input. Visualization of the signal during data acquisition is especially important for clinical applications because it is critical to ensure that the quality and quantity of data are adequate before the patient leaves the lab.

Despite our focus on fMCG, the ferrite shield is potentially useful for other biomagnetism measurements. As discussed above, it can help isolate the signal of interest from sources of biological, as well as environmental, interference. For instance, it can attenuate maternal and fetal cardiac interferences in fetal MEG and cardiac interference in magnetogastography and magnetoenterography studies. A recently demonstrated technique of simultaneously recording fMCG and fetal echocardiography [17]—magnetomechanical imaging—can also benefit from the ferrite shield to attenuate interferences from the ultrasound probe. This may allow simultaneous, real-time displays of fMCG, as well as pulsed Doppler and other echocardiography modalities, by connecting the fMCG to the ECG input of the ultrasound scanner. A significant limitation of the technique, however, is signal attenuation and/or distortion of the signal topography because of the ferrite shield. These effects are relatively small and inconsequential for fMCG, which utilizes the temporal information in the signals; however, they are likely unacceptable for source-localization applications, which require the highly accurate modeling of the signal topography.

The ferrite shield is a practical and cost-effective method of improving signal quality in comparison to the alternative methods of enhancing shielding performance mentioned in the introduction. It is simple and versatile. It can be used in conjunction with nearly any existing shield and can be easily put in place and removed as needed. Subjects report that the shield helps distribute the pressure on the abdomen and is more comfortable. Additionally, as they enter the shield feet first, their head is near the opening and they can see the outside, which minimizes the risk of claustrophobia. Using the ferrite shield may also allow the use of shorter person-sized shields, which can also help with this issue. A straightforward means of further improving the effectiveness of the method is to add a second layer of shielding. Mu-metal is much more widely used for magnetic shielding than ferrite because mu-metal is cheaper and has higher magnetic permeability. This suggests constructing a two-layer shield using ferrite for the inner shield and mu-metal for the outer shield, as implemented by Kornack and coworkers [16].

In conclusion, we demonstrated the use of a small ferrite shield as a simple, practical method of overcoming the high level of environmental interference present in fMCG recordings made using person-sized magnetic shields. The method allows users to take full advantage of newly available OPM sensors that record all three components of the signal and provide meaningful improvements in signal resolution and diagnostic capability. This can help make fMCG more widely available as a new technique for evaluating life-threatening fetal arrhythmia and other high-risk pregnancy conditions associated with abnormal fetal heart rate and rhythm.

## Figures and Tables

**Table 1:** Statistical results for fetal and maternal QRS amplitudes (fQRS and mQRS, respectively), noise amplitude, and signal-to-noise ratio (SNR) for horizontal and vertical transverse directions, with and without the ferrite shield. The * indicates *p*-value < 0.05.

Variables	Effects				*p*-Value		
	Horizontal Transverse		Vertical Transverse				
	Unshielded	Shielded	Unshielded	Shielded	Shield	Direction	Shield x Direction
fQRS (pT)	20.8 ± 1.1	11.1 ± 1.1	19.8 ± 1.2	13.4 ± 1.2	<0.0001 *	0.370	0.034 *
mQRS (pT)	22.8 ± 1.3	12.1 ± 1.3	23.0 ± 1.3	16.4 ± 1.3	<0.0001 *	0.072	0.098
Noise (pT)	8.0 ± 0.5	3.6 ± 0.5	7.1 ± 0.5	3.8 ± 0.5	<0.0001 *	0.313	0.119
SNR	2.7 ± 0.2	3.6 ± 0.2	2.9 ± 0.2	3.6 ± 0.2	<0.0001 *	0.081	0.313

## Data Availability

The datasets generated during and/or analyzed during the current study are available from the corresponding author upon reasonable request.
